# Constipation as a Drug-Related Adverse Effect in Patients with Hyperkalemia: Sodium Zirconium Cyclosilicate versus Conventional Potassium Binders

**DOI:** 10.3390/jcm12185971

**Published:** 2023-09-14

**Authors:** Yuki Hida, Teruhiko Imamura, Koichiro Kinugawa

**Affiliations:** Second Department of Medicine, University of Toyama, Toyama 930-0193, Japan

**Keywords:** potassium, heart failure, renal failure

## Abstract

(1) Background: Constipation is one of the most serious adverse effects of potassium-lowering agents and decreases patients’ quality of life. Sodium zirconium cyclosilicate (SZC) is a recently innovated potassium binder intended for patients with hyperkalemia. The impact of SZC on the worsening of constipation, as compared with conventional potassium binders, remains unknown. (2) Methods: Patients with hyperkalemia who continued SZC for over 3 months between July 2020 and May 2022 were included in this retrospective study. Patients who received other conventional potassium binders during the same period were included as a control group. Trends in the doses of anti-constipation agents during the 3-month therapeutic period were compared between the two groups as a surrogate for worsening constipation. (3) Results: A total of 50 patients (median age 74 years, 31 male) were included, consisting of 22 patients with SZC and 28 patients with other conventional potassium binders. All patients had hyperkalemia and chronic kidney disease at baseline. During the 3-month therapeutic period, serum potassium levels decreased significantly in both groups (*p* < 0.05 for both). The number of anti-constipation remained unchanged in the SZC group but tended to increase in the control group (*p* = 0.56 and *p* = 0.090, respectively). The total dose change in all anti-constipations was significantly lower in the SZC group than in the control group (*p* = 0.037). (4) Conclusions: Conventional potassium binders have a tendency to worsen constipation, whereas SZC may have the potential to improve hyperkalemia without worsening constipation. SZC may be recommended, particularly in elderly patients with ongoing or high-risk constipation.

## 1. Background

Hyperkalemia is associated with mortality and morbidity, especially in elderly patients with heart failure and/or chronic kidney disease [1,2]. Potassium binders are commonly used to treat hyperkalemia. However, one of the serious adverse effects of these drugs is constipation [3,4].

The exact mechanism is uncertain, but these polymers expand and stay in the intestine longer when deionized water is present. The expanded polymers stay and obstruct the intestine, resulting in persistent constipation [4,5]. Constipation should be appropriately managed, especially in patients with heart failure, because efforts to relieve constipation increase afterload and worsen heart failure [5]. The presence of constipation is also associated with the worsening of renal function and the development of chronic kidney disease [6]. On the contrary, both heart failure and chronic kidney disease independently worsen constipation, respectively. This mechanism is called cardio-enteric-renal syndrome [7]. The doses of anti-constipation agents are attempted to be increased, but refractory constipation sometimes persists and decreases patients’ quality of life [8]. Severe and long-standing constipation further worsens heart failure and chronic kidney disease, and vice versa.

Sodium zirconium cyclosilicate (SZC), a zirconium silicate compound that exchanges sodium and hydrogen for potassium and ammonium ions in the gastrointestinal tract [9], is clinically available. As a novel potassium binder, SZC lowers serum potassium levels and maintains them within the normal range with robust evidence [10,11,12,13,14]. In the previous in vivo study, the volume of SZC significantly and uniquely decreased when deionized water was added, probably because SZC is a non-polymer. This is one of the unique features of SZC compared with other conventional potassium binders, although it has not yet been clinically demonstrated [15].

Given all this, we hypothesized that SZC may have an advantage over conventional potassium binders in not worsening drug-related constipation, particularly in elderly patients with hyperkalemia who require such potassium-lowering therapy despite their constipation [16]. In this study, we compared the trend in the dose of anti-constipation agents during SZC therapy versus conventional potassium binder therapy as a surrogate for worsening constipation.

## 2. Methods

### 2.1. Patient Selection

Patients who continued SZC for over 3 months to treat their hyperkalemia (defined as serum potassium levels above 5.0 mEq/L) between July 2020 and May 2022 were retrospectively included in the SZC group. In the control group, patients who received other potassium-lowering agents, including sodium polystyrene sulfonate or calcium polystyrene sulfonate, for 3 months during the same period were included. Informed consent was obtained from all participants, and this study was approved by the institutional ethical board beforehand.

### 2.2. Study Design

Patients were followed for 3 months after the initiation of potassium binders (SZC in the SZC group and other potassium binders in the control group). The doses of anti-constipation agents during the 3-month therapeutic period were compared between the SZC group and the control group as a surrogate for worsening constipation (according to the world gastroenterology organization global guidelines) and were defined as a primary concern. When the dose of anti-constipation agents was increased, patients were assumed to have worsening constipation.

### 2.3. Potassium Binder Therapy

In principle, SZC was started for hyperkalemia at a loading dose of 30 g/day for 2 days, followed by a maintenance dose of 5–15 g/day according to the serum potassium levels at each visit. Other conventional potassium binders were also administered to treat hyperkalemia in a standard manner, and their doses were adjusted according to the serum potassium levels. During the therapeutic period, patients were educated to restrict potassium intake in a standard manner.

### 2.4. Anti-Constipation Agents

The doses of anti-constipation agents were adjusted according to the patient’s symptoms at the discretion of the attending physicians. Generally, osmotic laxatives and irritant laxatives were initially attempted. Their doses were titrated up when patients’ constipation persisted. Additional secretagogues were considered for refractory constipation.

For the assessment of anti-constipation use, their number was counted. For example, when a patient used magnesium oxide and sennoside, the medication number was counted as two, regardless of their doses. The total dose change during the 3-month therapeutic period was also assessed. For example, in the same patient, the dose of magnesium oxide was increased (i.e., a dose change was “+1”) and the dose of sennoside was decreased (i.e., a dose change was “−1”). The total dose change was calculated as zero. An initiation of a new drug was assumed as an increase, and a termination of the drug was assumed as a decrease.

### 2.5. Other Clinical Variables

Demographics, comorbidity, laboratory, and medication data at baseline, when potassium binders were initiated, were retrieved. Laboratory and medication data were also obtained at the 3-month follow-up.

### 2.6. Statistical Protocol

Statistical analyses were conducted using SPSS Statistics 23 (SPSS Inc., Armonk, IL, USA). Two-tailed *p*-values < 0.05 were assumed to be statistically significant. All continuous variables were assumed to be non-parametric data considering the small sample size. Continuous variables were stated as median and interquartile (25% interquartile, 75% interquartile). Categorical variables were presented as numbers and percentages. Trends in clinical parameters during the observation period were compared using the Wilcoxon signed-rank test for the continuous variables and the McNemar test for the categorical variables. A logistic regression analysis was conducted to evaluate the impact of no SZC use (i.e., the use of other conventional potassium binders) on the positive total dose change. The impact of SZC use (versus conventional potassium binder) on worsening constipation was adjusted for age and heart failure, which may also have a significant impact on constipation.

## 3. Results

### 3.1. Baseline Characteristics

A total of 50 patients were included in this retrospective study (Table 1). The median age was 74 (68, 84) years old, and 31 (62%) were male. Twenty-two patients received SZC and were assigned to the SZC group. Twenty-eight patients received other conventional potassium binders (13 sodium polystyrene sulfonate and 15 calcium polystyrene sulfonate) and were assigned to the control group. All patients continued these potassium binders (i.e., SZC or other conventional ones) for three months to manage their hyperkalemia. All patients had chronic kidney disease, and 21 (42%) had heart failure.

### 3.2. Trends in Clinical Data in the SZC Group

During 3-month SZC therapy, serum potassium levels decreased significantly from 5.4 (5.0, 5.8) mEq/L to 4.4 (4.2, 4.9) mEq/L (*p* < 0.001) (Table 2). No patients had drug-related hypokalemia that disturbed SZC continuation. The prescription rates of renin–angiotensin system inhibitors remained unchanged, and those of mineralocorticoid receptor antagonists tended to decrease. The doses of each anti-constipation agent remained unchanged, indicating no worsening of constipation (*p* > 0.05 for all).

### 3.3. Trends in Clinical Data in the Control Group

During 3-month conventional potassium binder therapy, serum potassium levels decreased significantly from 5.5 (5.3, 5.8) mEq/L to 5.0 (4.6, 5.2) mEq/L (*p* = 0.006) (Table 3). The prescription rates of renin–angiotensin system inhibitors and mineralocorticoid receptor antagonists remained unchanged (*p* > 0.05 for both). The doses of anti-constipation agents remained unchanged, except for magnesium oxide, whose dose increased significantly (*p* = 0.040).

### 3.4. Comparison in Anti-Constipation between the Two Groups

**Figure 1:**
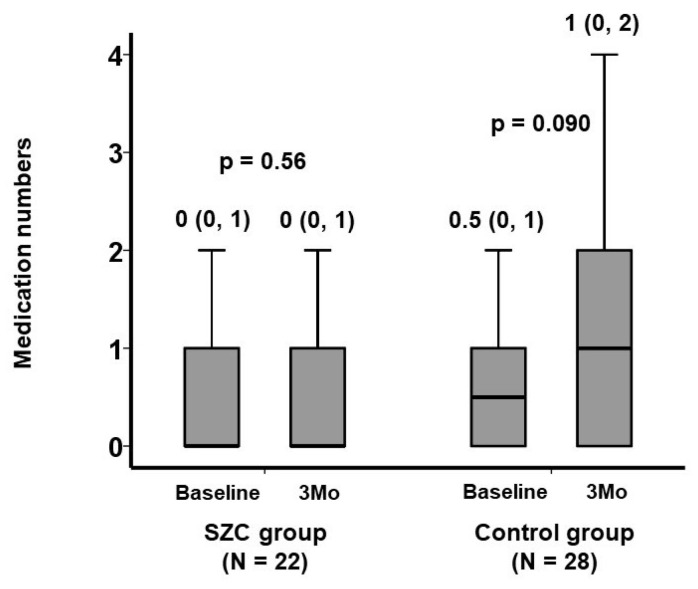
Trends in the number of anti-constipation agents during the 3-month therapeutic period. The number of anti-constipation agents remained unchanged in the SZC group whereas those in the control group tended to increase during 3-month therapeutic period. The number of each anti-constipation agent was counted regardless of its dose. The trends were assessed using the Wilcoxon signed-rank test.

**Figure 2:**
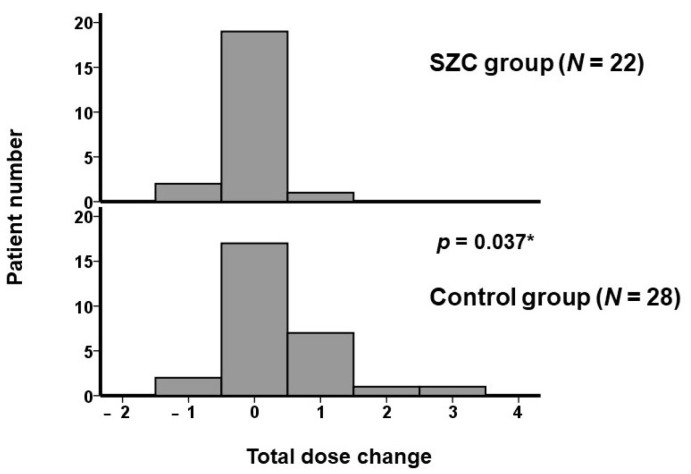
Distribution of total dose change during the 3-month therapeutic period in the two groups. Most patients in the SZC group had a total dose change of zero, whereas some patients had 1–3 total dose changes in the control group. The total dose change was significantly lower in the SZC group than in the control group. For example, when a patient had an increase in dose of magnesium oxide (dose change was defined as “+1”) and a decrease in sennoside (dose change was defined as “−1”), a total dose change was calculated as zero. * *p* < 0.05 by Wilcoxon signed-rank test.

The numbers of anti-constipation agents remained unchanged in the SZC group (*p* = 0.56), whereas those in the control group tended to increase from 0.5 (0, 1) to 1 (0, 2) (*p* = 0.090) (Figure 1). There were 2 dose increases and 1 dose decrease in the SZC group, whereas there were 13 dose increases and 3 dose decreases in the control group. The distribution of total dose change in each patient is displayed in Figure 2. The total dose change was significantly lower in the SZC group (i.e., the dose of anti-constipation agent was less up-titrated) than in the control group (*p* = 0.037). In other words, constipation rarely worsened during SZC therapy, whereas constipation tended to worsen during conventional potassium binder therapy.

A total of 10 patients had a positive total dose change (i.e., the doses of anti-constipation agents were increased overall). The use of other conventional potassium binders was associated with a positive total dose change with an unadjusted odds ratio of 9.95 (95% confidence interval 1.15–86.0, *p* = 0.037) and an odds ratio of 8.70 (95% confidence interval 0.96–78.6, *p* = 0.054) adjusted for age and the presence of heart failure.

## 4. Discussion

In this study, we included patients who received SZC for three months as an SZC group and those who received other conventional potassium binders for three months as a control group, both of which were administered to treat hyperkalemia. The trend of anti-constipation agents during the 3-month therapeutic period was compared between the two groups as a surrogate for worsening constipation (i.e., up-titration of anti-constipation agents was assumed to worsen constipation).

All participants had chronic kidney disease, and almost half of them had heart failure. Serum potassium levels decreased significantly, regardless of the types of potassium binders, and no patients had drug-related hypokalemia that disturbed medication continuation. The number of anti-constipation agents remained unchanged in the SZC group, whereas those in the control group tended to increase. The total dose change was significantly lower in the SZC group compared with the control group (i.e., the dose of anti-constipation agents was less up-titrated during SZC therapy than during conventional potassium binder therapy).

### 4.1. Impact of SZC and Other Potassium Binders on Hyperkalemia

Oral potassium binders have been clinically utilized to treat chronic hyperkalemia. However, these conventional potassium binders do not have robust evidence to improve hyperkalemia appropriately and immediately or to maintain serum potassium levels within the acceptable range (i.e., approximately between 3.5 mEq/L and 5.0 mEq/L) [17]. Given the lack of large-scale studies involving these conventional drugs, the detailed incidence of drug-related adverse events remained uncertain.

SZC has been shown to treat hyperkalemia immediately and to maintain serum potassium levels in the normal range for months with acceptably minimal drug-related adverse effects regardless of the dependence on hemodialysis [10,11,12,13,14]. Although it is not a primary concern of this study, hyperkalemia was improved in this study regardless of the types of drugs. Of note, serum potassium levels fluctuated around a relatively higher range (i.e., around 5.0 mEq/L) in the control group, whereas serum potassium levels were strictly controlled within the normal range in the SZC group.

### 4.2. Potassium Binders and Worsening Constipation

All participants had chronic kidney disease, and approximately half had heart failure, both of which often complicate hyperkalemia via direct and indirect mechanisms known as cardio-enteric-renal syndrome [7]. It is well known that conventional potassium binders are associated with chronic constipation and any other digestive symptoms because these agents expand in the gastrointestinal tract [4]. Our patients with conventional potassium binders consistently had worsening constipation, which required up-titration of anti-constipation agents.

Patients with heart failure and/or chronic kidney disease are at high risk of constipation [7]. Both cardiac cachexia and visceral congestion due to heart failure decrease bowel motility. The use of diuretics to treat systemic/pulmonary congestion causes hard stools. Uremia due to renal impairment disrupts the stability of gut bacteria. Potassium intake restriction to treat concomitant hyperkalemia decreases the intake of dietary fiber and further worsens constipation.

Constipation should be treated, particularly in these cohorts, because it worsens these comorbidities. For example, the effort against constipation increases the afterload on the left ventricle [5]. Persistent constipation unstabilizes gut bacteria and progresses chronic kidney disease [6]. The worsening of these comorbidities further progress constipation.

### 4.3. SZC and Worsening Constipation

In the previous in vivo study, the absolute volume of SZC significantly and uniquely decreased when deionized water was added, probably because SZC is a non-polymer. This is one of the unique features of SZC compared with other conventional potassium binders [15]. Furthermore, the dose of SZC that we prescribed was only 5–15 g per day as compared to other conventional potassium binders: 25 g of calcium polystyrene sulfonate and 30 g of sodium polystyrene sulfonate. In the HARMONIZE phase III trial, the incidence of constipation was below 10% during SZC therapy [14]. In our study, the doses of anti-constipation agents remained unchanged during SZC therapy, although the included patients were at high risk of constipation (i.e., elderly patients with multiple comorbidities). Although we did not assess the degree of constipation directly, constipation may not worsen during SZC therapy as opposed to other conventional potassium binders [18].

### 4.4. Clinical Implications

Although further, longer studies are warranted, SZC appears to be relatively protective against worsening constipation compared with conventional potassium binders. Patients with persistent and/or progressive constipation may be good candidates to switch from conventional potassium binders to SZC. Patients with heart failure and/or chronic kidney disease may also be good candidates for SZC because they are at high risk of constipation, which further worsens these comorbidities. Further studies are warranted to clarify the relationship between potassium binders, constipation, and clinical outcomes.

### 4.5. Limitations

This study is a proof-of-concept, including a small sample size likely due to the limited prescription of SZC to treat hyperkalemia so far. The amount and dose of anti-constipation agents in the SZC group remained statistically unchanged, but this does not necessarily indicate “similarity” due to the small sample size. Recently, it has been recommended to assume continuous variables as non-parametric data if their sample size is small, regardless of their distribution (i.e., normal versus non-normal). Thus, due to the small sample size, we assumed all continuous variables in this study to be non-parametric data.

Given the nature of the retrospective study, we focused on the prescription of anti-constipation agents as a surrogate for worsening constipation instead of a detailed assessment of constipation-related symptoms. There are a variety of scales to assess the severity of constipation, although most of them are subjective. The data on anti-constipation may have an advantage in its objectiveness over constipation-related symptoms. The world gastroenterology organization global guidelines define constipation as the use of any anti-constipation agent [19]. On the contrary, the prescription of anti-constipation agents was at the discretion of attending physicians.

Due to the retrospective nature of this study, baseline characteristics were not completely matched between the two groups. Other unevaluated clinical parameters may also be different between the two groups. We attempted to adjust for some of them, but any other unevaluated potential confounders may have affected the findings. Patients were asked to restrict potassium intake in a standard manner. Nevertheless, excessive potassium restriction may have reduced the intake of dietary fibers and worsened constipation in some patients. The actual amount of potassium intake was not counted.

## 5. Conclusions

SZC may have an advantage over conventional potassium binders in preventing the worsening of drug-related constipation in elderly patients with hyperkalemia accompanying chronic kidney disease. Further studies are warranted for constructing optimal protocol selection for SZC therapy rather than conventional potassium binders.

## Figures and Tables

**Table 1:** Baseline characteristics.

	Total (N = 50)	SZC Group (N = 22)	Control Group (N = 28)	*p* Value
Demographics				
Age, years	74 (68, 84)	78 (70, 84)	73 (67, 81)	0.20
Male sex	31 (62%)	13 (59%)	18 (64%)	0.47
Body mass index	22.2 (20.7, 24.0)	21.2 (18.8, 22.6)	23.6 (21.3, 24.4)	0.087
Comorbidity				
Heart failure	21 (42%)	13 (59%)	8 (29%)	0.030 *
Diabetes mellitus	29 (58%)	13 (59%)	16 (57%)	0.56
Atrial fibrillation	14 (28%)	9 (41%)	5 (18%)	0.069
Coronary heart disease	10 (20%)	6 (27%)	4 (14%)	0.25
History of stroke	2 (4%)	2 (9%)	0 (0%)	0.10
Chronic kidney disease	50 (100%)	22 (100%)	28 (100%)	1.0
Stage G3a/G3b/G4/G5	8/11/22/9	4/7/7/4	4/4/15/5	0.65
Hemodialysis	4 (8%)	2 (9%)	2 (7%)	0.80

Continuous variables are stated as median (25% interquartile, 75% interquartile) and compared between the two groups using Mann–Whitney U test. Categorical variables are stated as numbers and percentages and compared between the two groups using Fischer’s exact test. * *p* < 0.05.

**Table 2:** Trends in clinical parameters during 3-month SZC therapy (N = 22).

	Baseline	Three Months Later	*p* Value
Laboratory data			
Hemoglobin, g/dL	11.0 (9.4, 11.3)	11.2 (10.1, 11.8)	0.042 *
Serum albumin, g/dL	3.7 (3.2, 3.9)	3.7 (3.4, 4.0)	0.036 *
Serum sodium, mEq/L	139 (136, 141)	140 (137, 141)	0.25
Serum potassium, mEq/L	5.4 (5.0, 5.8)	4.4 (4.2, 4.9)	<0.001 *
Estimated glomerular filtration rate, mL/min/1.73 m^2^	30.3 (19.2, 43.2)	32.1 (18.4, 48.0)	0.41
Medications			
Renin–angiotensin system inhibitor	20 (91%)	20 (91%)	1.0
Mineralocorticoid receptor antagonist	15 (68%)	9 (41%)	0.070
Furosemide, mg/day	0 (0, 0)	0 (0, 20)	0.67
Magnessium oxide, mg/day	0 (0, 0.66)	0 (0, 0)	0.66
Sennoside, mg/day	0 (0, 0)	0 (0, 0)	0.32
Movicol, mg/day	0 (0, 0)	0 (0, 0)	1.0
Lubiprost, mg/day	0 (0, 0)	0 (0, 0)	0.32
Erobikistat, mg/day	0 (0, 0)	0 (0, 0)	1.0

Continuous variables are stated as median (25% interquartile, 75% interquartile) and their trends were assessed using Wilcoxon signed-rank test. Categorical variables are stated as numbers and percentages, and their trends were assessed using McNemar test. * *p* < 0.05.

**Table 3:** Trends in clinical parameters during 3-month therapy using conventional potassium binders (N = 28).

	Baseline	Three Months Later	*p* Value
Laboratory data			
Hemoglobin, g/dL	9.6 (8.9, 10.8)	11.1 (9.5, 11.8)	0.37
Serum albumin, g/dL	3.7 (3.5, 3.9)	3.6 (3.2, 3.7)	1.0
Serum sodium, mEq/L	139 (137, 141)	139 (138, 141)	0.38
Serum potassium, mEq/L	5.5 (5.3, 5.8)	5.0 (4.6, 5.2)	0.006 *
Estimated glomerular filtration rate, mL/min/1.73 m^2^	23.7 (19.3, 30.8)	15.4 (10.7, 20.3)	0.001 *
Medications			
Renin–angiotensin system inhibitor	19 (68%)	21 (75%)	0.73
Mineralocorticoid receptor antagonist	3 (11%)	2 (7%)	1.0
Furosemide, mg/day	0 (0, 0)	0 (0, 0)	0.58
Magnessium oxide, mg/day	0 (0, 0)	0 (0, 0.66)	0.040 *
Sennoside, mg/day	0 (0, 0)	0 (0, 12)	0.75
Movicol, mg/day	0 (0, 0)	0 (0, 0)	0.32
Lubiprost, mg/day	0 (0, 0)	0 (0, 0)	0.16
Erobikistat, mg/day	0 (0, 0)	0 (0, 0)	0.32

Continuous variables are stated as median (25% interquartile, 75% interquartile) and their trends were assessed using Wilcoxon signed-rank test. Categorical variables are stated as numbers and percentages, and their trends were assessed using the McNemar test. * *p* < 0.05.

## Data Availability

Data are available upon reasonable request from the corresponding author.

## References

[1] Sarwar C.M., Papadimitriou L., Pitt B., Pina I., Zannad F., Anker S.D., Gheorghiade M., Butler J. (2016). Hyperkalemia in Heart Failure. J. Am. Coll. Cardiol..

[2] Seliger S.L. (2019). Hyperkalemia in patients with chronic renal failure. Nephrol. Dial. Transplant..

[3] Natale P., Palmer S.C., Ruospo M., Saglimbene V.M., Strippoli G.F. (2020). Potassium binders for chronic hyperkalaemia in people with chronic kidney disease. Cochrane Database Syst. Rev..

[4] Narita Y., Fukumoto K., Fukunaga M., Kondo Y., Ishitsuka Y., Jono H., Irie T., Saito H., Kadowaki D., Hirata S. (2020). Comparative Study of Constipation Exacerbation by Potassium Binders Using a Loperamide-Induced Constipation Model. Int. J. Mol. Sci..

[5] Ishiyama Y., Hoshide S., Mizuno H., Kario K. (2019). Constipation-induced pressor effects as triggers for cardiovascular events. J. Clin. Hypertens..

[6] Sumida K., Molnar M.Z., Potukuchi P.K., Thomas F., Lu J.L., Matsushita K., Yamagata K., Kalantar-Zadeh K., Kovesdy C.P. (2017). Constipation and Incident CKD. J. Am. Soc. Nephrol..

[7] Sumida K., Kovesdy C.P. (2019). The gut-kidney-heart axis in chronic kidney disease. Physiol. Int..

[8] Sumida K., Yamagata K., Kovesdy C.P. (2020). Constipation in CKD. Kidney Int. Rep..

[9] Hoy S.M. (2018). Sodium Zirconium Cyclosilicate: A Review in Hyperkalaemia. Drugs.

[10] Packham D.K., Rasmussen H.S., Lavin P.T., El-Shahawy M.A., Roger S.D., Block G., Qunibi W., Pergola P., Singh B. (2015). Sodium zirconium cyclosilicate in hyperkalemia. N. Engl. J. Med..

[11] Spinowitz B.S., Fishbane S., Pergola P.E., Roger S.D., Lerma E.V., Butler J., von Haehling S., Adler S.H., Zhao J., Singh B. (2019). Sodium Zirconium Cyclosilicate among Individuals with Hyperkalemia: A 12-Month Phase 3 Study. Clin. J. Am. Soc. Nephrol..

[12] Zannad F., Hsu B.G., Maeda Y., Shin S.K., Vishneva E.M., Rensfeldt M., Eklund S., Zhao J. (2020). Efficacy and safety of sodium zirconium cyclosilicate for hyperkalaemia: The randomized, placebo-controlled HARMONIZE-Global study. ESC Heart Fail..

[13] Roger S.D., Spinowitz B.S., Lerma E.V., Singh B., Packham D.K., Al-Shurbaji A., Kosiborod M. (2019). Efficacy and Safety of Sodium Zirconium Cyclosilicate for Treatment of Hyperkalemia: An 11-Month Open-Label Extension of HARMONIZE. Am. J. Nephrol..

[14] Kosiborod M., Rasmussen H.S., Lavin P., Qunibi W.Y., Spinowitz B., Packham D., Roger S.D., Yang A., Lerma E., Singh B. (2014). Effect of sodium zirconium cyclosilicate on potassium lowering for 28 days among outpatients with hyperkalemia: The HARMONIZE randomized clinical trial. JAMA.

[15] Rafique Z., Peacock W.F., LoVecchio F., Levy P.D. (2015). Sodium zirconium cyclosilicate (ZS-9) for the treatment of hyperkalemia. Expert Opin. Pharmacother..

[16] Imamura T., Kinugawa K. (2022). Successful Conversion from Conventional Potassium Binder to Sodium Zirconium Cyclosilicate in a Patient with Refractory Constipation. Medicina.

[17] Palmer B.F., Carrero J.J., Clegg D.J., Colbert G.B., Emmett M., Fishbane S., Hain D.J., Lerma E., Onuigbo M., Rastogi A. (2021). Clinical Management of Hyperkalemia. Mayo Clin. Proc..

[18] Imamura T., Fujioka H., Narang N., Kinugawa K. (2023). Impact of sodium zirconium cyclosilicate therapy on nutrition status in patients with hyperkalemia. J. Clin. Med..

[19] Lindberg G., Hamid S.S., Malfertheiner P., Thomsen O.O., Fernandez L.B., Garisch J., Thomson A., Goh K.L., Tandon R., Fedail S. (2011). World Gastroenterology Organisation global guideline: Constipation—A global perspective. J. Clin. Gastroenterol..

